# A time trade-off study in the UK, Canada and the US to estimate utilities associated with the treatment of haemophilia

**DOI:** 10.1186/s12955-024-02311-5

**Published:** 2024-11-13

**Authors:** Anna Okkels, Cecilie Yssing, Michael Lyng Wolden, Mohd Nawi Wahid

**Affiliations:** 1 EY Godkendt Revisionspartnerselskab, Frederiksberg, 2000 Denmark; 2 Novo Nordisk, Vandtårnsvej 108-110, Søborg, 2860 Denmark; 3 Novo Nordisk A/S, Zurich, Switzerland

**Keywords:** Canada, Caregiver, Haemophilia, Health-related quality of life, Preferences, Time trade-off study, Treatment, UK, US, Utility value

## Abstract

**Introduction:**

Haemophilia is a rare bleeding disorder caused by a deficient or absent clotting factor, leading to frequent bleeding. Multiple intravenous (IV) infusions have been the standard prophylactic treatment; however, newer treatment options involve less frequent subcutaneous (SC) injections. To inform future health economic evaluations, this study applied the time trade-off (TTO) method for estimation of utilities associated with haemophilia treatment for both people with the disease and potential caregivers.

**Methods:**

Using the TTO method, utilities were estimated through two online surveys distributed in the UK, Canada and the US. In survey 1 (S1), adults from the general population aged 18 years and above evaluated health states as if they were living with haemophilia themselves and were receiving treatment for the condition. In survey 2 (S2), adults from the general population with a child under the age of 15 years evaluated health states as if they were treating their child for haemophilia. The surveys assessed the following treatment aspects: frequency of treatment, treatment device and injection site reactions.

**Results:**

In total, 812, 739 and 703 respondents completed S1 and 712, 594 and 527 completed S2 in the UK, Canada and the US, respectively. In both surveys, the treatment device was associated with the largest impact on utilities for both people with haemophilia and caregivers. Monthly SC injections with a prefilled pen-device were associated with a significant utility gain compared with SC injections with a syringe and IV infusions. In S1, a lower treatment frequency was preferred in all three countries, while in S2, a lower treatment frequency was preferred only in the UK. Avoiding injection site reactions was associated with a significant utility gain in both surveys, but only in the UK and Canada.

**Conclusions:**

The study suggests that the administration of haemophilia treatment in particular has an impact on utilities for both people and caregivers living with the disease. Thus, less complex and time-consuming treatment devices are expected to improve health-related quality of life. This can be further modified additively by less frequent administration. These results can inform future health economic analyses of haemophilia and haemophilia treatment.

**Supplementary Information:**

The online version contains supplementary material available at 10.1186/s12955-024-02311-5.

## Background

Haemophilia is a rare hereditary bleeding disorder caused by a deficient or absent clotting factor in the blood, resulting in inadequate blood coagulation. Accounting for 80–85% of all haemophilia cases, haemophilia A is the most frequent type [1]. The condition is most present in males, and the worldwide prevalence of haemophilia A among males is 17.1 per 100,000. Of these, 6.0 per 100,000 are severe cases [2, 3]. In the US, the prevalence of haemophilia A at birth has been reported to be 17.9 per 100,000 among males, whereas it has been reported to be 19.1 and 24.6 per 100,000 in Canada and the UK, respectively [3, 4].

People living with haemophilia experience frequent episodes of bleeding, particularly in joints and muscles, causing destruction of articular structures, impaired function and pain [1, 5, 6]. Additionally, haemophilia impacts life expectancy and can result in comorbidities such as cardiovascular and metabolic disease, renal disease, and cancer [1, 3]. Due to its symptoms and comorbidities, haemophilia severely impacts health-related quality of life (HRQoL) [7, 8]. Whereas pain and mobility problems have been reported as the main difficulties among people with haemophilia, sudden or unexpected bleeding were reported as the main difficulties for caregivers [9].

Historically, prophylactic treatment of haemophilia has involved multiple weekly intravenous (IV) infusions, but newer treatment options involve less frequent subcutaneous (SC) injections [10]. Prophylactic treatment increases HRQoL, decreases the number of bleeding episodes and damage to the joints, and reduces the number of problems with mobility, pain and discomfort [7, 11]. Despite the treatment benefits, previous studies have shown a high perceived treatment burden, e.g. due to the complicated and time-consuming process. The perceived burden seems to be even heavier among people on prophylactic treatment than among people using episodic treatment only [1, 12–15]. Additionally, the treatment burden has been reported as substantial among caregivers, who experience both practical and emotional challenges [15, 16].

To inform health economic evaluations, several health technology assessment (HTA) agencies require estimation of utilities when measuring a specific condition’s impact on HRQoL. Some HTA agencies also recommend including utilities for caregivers [17]. Generic measures, e.g. EQ-5D, are often the preferred tool for utility generation. However, when measuring HRQoL for rare diseases or when estimating treatment process utilities, vignettes-based approaches, such as the time trade-off (TTO) method, are acknowledged by several HTA agencies and recommended as a relevant method [18–20]. The TTO method enables evaluation of more disease-specific conditions and offers a unique opportunity to evaluate the impact of different treatment aspects on HRQoL, including, e.g. mode and frequency of administration, treatment convenience [17, 18, 21].

The aim of this study was to estimate utilities associated with haemophilia treatment for people with the disease and potential caregivers. This was investigated by evaluating different aspects of haemophilia treatment using the TTO method in the UK, Canada and the US.

## Methods

### Study population

In this study, two TTO surveys were developed to estimate utilities associated with haemophilia treatment (details in the Appendix). Based on the recommendations of several HTA agencies, the study population for both surveys was recruited from the general population [17–19, 22]. Respondents were recruited through existing email panels and were awarded points equivalent to EUR/CAD/USD 1–2 for their participation.

Survey 1 (S1) was designed to estimate utilities for people with haemophilia, and survey 2 (S2) was designed to estimate utilities for caregivers of children with haemophilia, i.e. the impact on caregivers’ HRQoL when being responsible for treating a child with haemophilia.

The inclusion criteria were consent to participate and at least 18 years of age. Since haemophilia mainly exists among men, only male respondents were included in S1. In S2, only respondents with at least one child under the age of 15 years were included. This followed recommendations by Powell et al. (2021) [23] and increased relatability for respondents when answering questions.

No sensitive information was collected or revealed in the surveys, and all answers were made anonymous. The surveys were conducted according to the codes of conduct of the Market Research Society and followed the applicable ESOMAR guidelines. Ethical review board approval was not required, since the study was not a clinical trial, it did not include patients, and it did not gather biological or human samples or identifiable personal information.

### Description of disease and definition of health states

Based on findings from previous focus group interviews in the UK and the US including 22 people with haemophilia and 16 caregivers (not published) as well as insights from medical experts, descriptions of haemophilia and haemophilia treatment were included in the surveys.

**Fig. 1:**
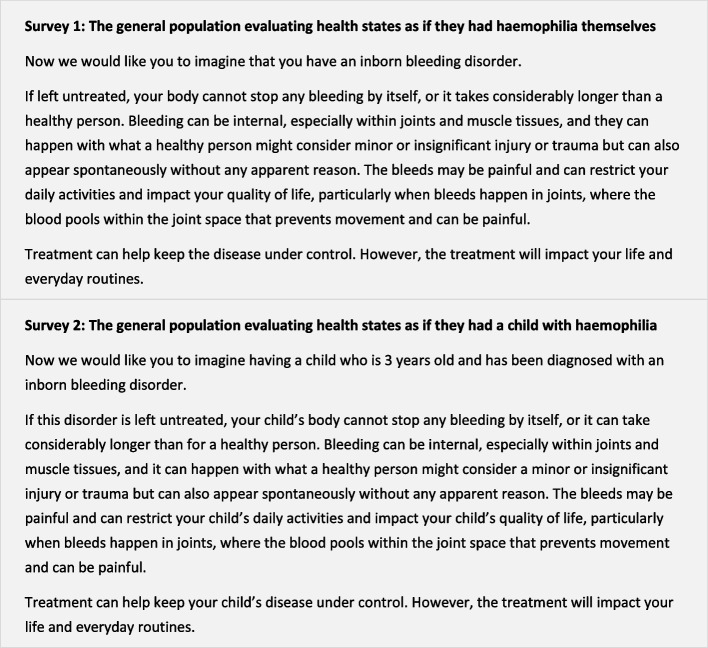
Description of haemophilia in the two surveys

At the beginning of each survey, respondents were introduced to haemophilia; however, to minimise bias in answers, the disease name was never mentioned. Instead, respondents were asked to imagine either having an inborn bleeding disorder (S1) or having a 3-year-old child with an inborn bleeding disorder (S2) (Fig. 1).

Following the disease introduction, three aspects of haemophilia treatment were presented in each survey: 1) frequency of treatment, 2) treatment device and 3) injection site reactions (Table 1). In order to make the results as realistic as possible, the descriptions of the treatment aspects were based on product information from existing treatments and a definition of injection site reactions from a previous phase 3 clinical trial [24]. Additionally, the final descriptions were validated by medical experts. The description of treatment aspects is included in the Appendix.

**Table 1:** Overview of evaluated aspects in the treatment of haemophilia

Aspect	Opportunities	Description
**Frequency of treatment**	Weekly	Once a week
	Biweekly	Once every second week
	Monthly	Once every fourth week
**Treatment device**	Prefilled pen-device for SC injections	An injection under the skin with a prefilled pen device The process takes around one minute and requires four steps
	Syringe for SC injections	An injection under the skin with a syringe The process takes around five minutes and requires 16 steps
	Syringe for IV infusions	An infusion in the vein with a syringe The process takes around 10 min and requires 12 steps
**Injection site reactions**	No injection site reactions	The treatment causes no injection site reactions
	Injection site reactions	The treatment causes injection site reactions which do not require medical intervention

*SC* Subcutaneous

*IV* Intravenous

To elicit utilities associated with the three treatment aspects, nine health states describing different hypothetical haemophilia treatment options were designed (Table 2). In S1, health states were described as permanent conditions that respondents should imagine living with for their remaining lifespan. In S2, health states were described as conditions lasting for 11 years in which respondents should imagine treating their child from the age of 3 until the age of 14. Since previous findings in the literature indicate that the perspective introduced to respondents in vignettes-based research has an impact on respondents’ evaluations – especially if the perspective includes imagination of a child [23, 25–27] – the age interval of the child presented in S2 was chosen based on earlier research [28]. Since different treatment aspects usually are less impactful than the efficacy, safety or symptom profile associated with a specific treatment, the impact of these factors was minimised by describing all health states as treatments that keep the disease under control. Examples of how health states were presented for respondents are provided in Table A1 and Table A2.

**Table 2:** Overview of health states evaluated in the two surveys

Health state	1	2	3	4	5	6	7	8	9
**Frequency of treatment**	Monthly	Weekly	Biweekly	Monthly	Monthly	Monthly	Weekly	Weekly	Monthly
**Treatment device**	Pen	Pen	Pen	Syringe	Syringe	2 × syringe	Syringe	IV	IV
**Injection site reactions**	Never	Never	Never	Always	Never	Never	Never	Never	Never

*Included in block 1: Health states 1, 2, 3*

*Included in block 2: Health states 1, 4, 5, 6, 7*

*Included in block 3: Health states 1, 5, 8, 9*

### Survey design

The online surveys were programmed using a commercial survey software package (SurveyXact).

To make the questions as realistic as possible, time horizons presented for respondents varied depending on their remaining life expectancy (details in the Appendix) [29, 30]. Thus, in both surveys, respondents were asked to trade some of their own remaining life years to avoid living in an impaired health state in which they were treating themselves (S1) or their child (S2).

A number of features were built into the surveys to ensure that respondents understood and accepted the premise of the method. First, a warm-up TTO question was implemented as the first TTO question. This followed recommendations in the literature and helped familiarise respondents with the concept [31]. These results were not included in further analyses. Second, respondents’ trading behaviour was screened carefully if they were willing to trade the largest possible amount of life or if they were not willing to trade at all. In these cases, respondents were excluded from the analyses if they reported reasons for the behaviour that compromised the TTO method (details in the Appendix). Third, each survey was divided into three blocks containing three to five of the nine investigated health states (Table 2). To avoid fatigue, respondents were randomised to one block and received only questions about the health states included in that block. Finally, to ensure that results were not affected by the order of the questions, that was randomised as well.

A pilot study was conducted in the UK to test the survey functionality. Since it did not show unexpected or unusual results, the main data collection was initiated in the UK, Canada and the US without further changes and was conducted in August and September 2023.

### Statistical analysis

To estimate utilities associated with haemophilia treatment, average utilities for each of the nine health states were calculated. Thereafter, utility differences per year associated with the three investigated treatment aspects were calculated as the difference between the utilities of two health states (details in the Appendix). Only differences between utilities derived from the same block were estimated.

To enhance result reliability and reduce susceptibility to extreme data points, the analysis excluded the most extreme 5% of values (2.5% from each end). In a sensitivity analysis, all values were included. Nonparametric bootstrapping with 10,000 iterations was employed to simulate standard errors and confidence intervals (CIs) and to assess the difference between the parameters. All statistical analyses were conducted using SAS version 9.4 statistical software.

All results are presented as utility gains or disutilities per year associated with the investigated aspects of haemophilia treatment. Results assessed to be the most relevant for future economic evaluations are presented in this article, with additional results and results of sensitivity analysis presented in the Appendix (Tables A3 – A6).

## Results

### Study population

**Fig. 2:**
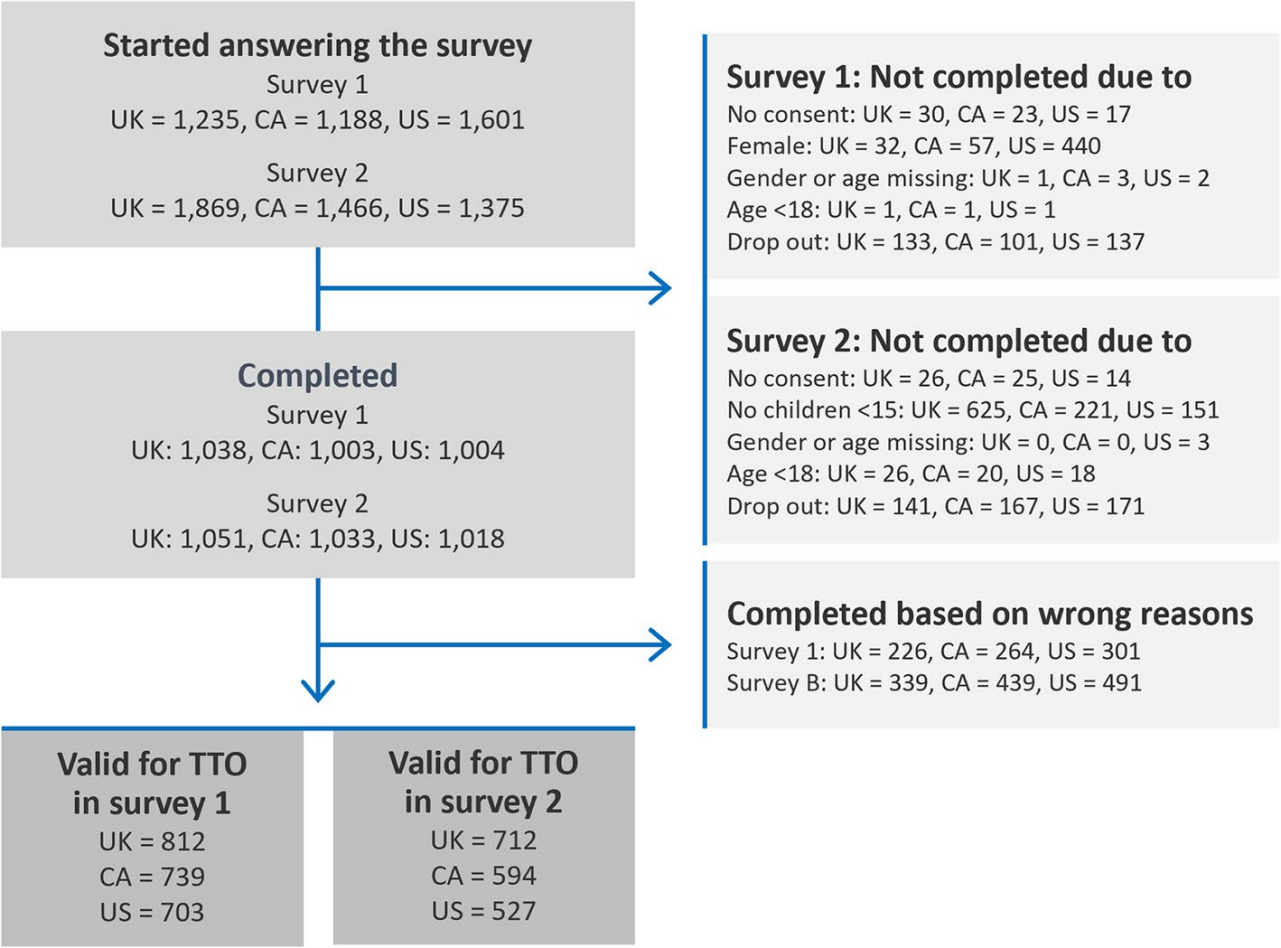
Respondent flowchart. Note: Reasons that result in exclusion from the survey included trading/not trading due to ethical or religious beliefs or due to not understanding the questions

In total, 1,038, 1,003 and 1,004 subjects completed S1 in the UK, Canada and the US, respectively. After exclusion of respondents who reported reasons for their behaviour that compromised the TTO method (wrong reasons for trading), 812, 739 and 703 respondents in the UK, Canada and the US, respectively, were valid for inclusion in the analysis of S1 (Fig. 2). In S2, 1,051, 1,033 and 1,018 subjects completed the survey in the UK, Canada and the US, respectively; however, 339, 439 and 491 were excluded due to reporting reasons for behaviour that compromised the TTO method (wrong reasons for trading). Thus, 712, 594 and 527 respondents were included in the analysis of S2 (Fig. 2).

In S1, the populations were similar across the UK, Canada and the US. The average age of respondents was almost the same, and the majority were in full-time employment and had a bachelor’s degree or higher. In the UK, the majority were married/living with a partner and had at least one child, and almost the same number in both Canada and the US either were married/living with a partner and had at least one child or were single and without children (Table 3).

**Table 3:** Characteristics of study population

	**Survey 1**			**Survey 2**		
	**UK**	**Canada**	**US**	**UK**	**Canada**	**US**
**Total, n**	812	739	703	712	594	527
**Gender, n (%)**						
Female	-	-	-	386 (54)	391 (66)	217 (41)
Male	812 (100)	739 (100)	703 (100)	326 (46)	203 (34)	310 (59)
**Age, n (%)**						
< 30	94 (12)	90 (12)	58 (8)	74 (10)	61 (10)	93 (18)
30–39	180 (22)	149 (20)	145 (21)	310 (44)	250 (42)	261 (50)
40–49	101 (12)	124 (17)	138 (20)	212 (30)	216 (36)	127 (24)
50–59	180 (22)	150 (20)	103 (15)	103 (14)	55 (9)	41 (8)
60–69	135 (17)	142 (19)	109 (16)	11 (2)	12 (2)	5 (1)
70 +	122 (15)	84 (11)	150 (21)	2 (< 1)	0 (0)	0 (0)
**Employment status, n (%)**						
Employed full-time	453 (56)	357 (48)	266 (38)	457 (64)	353 (59)	359 (68)
Part-time (< 32 h week)	45 (6)	69 (9)	72 (10)	107 (15)	67 (11)	47 (9)
Self-employed	52 (6)	40 (5)	50 (7)	54 (8)	45 (8)	47 (9)
Not employed	28 (3)	32 (4)	56 (8)	38 (5)	71 (12)	38 (7)
Retired	186 (23)	168 (23)	211 (30)	3 (< 1)	10 (2)	7 (1)
Student	11 (1)	32 (4)	10 (1)	4 (1)	9 (2)	5 (1)
Permanent disability	27 (3)	29 (4)	31 (4)	18 (3)	11 (2)	16 (3)
Other	6 (1)	6 (1)	5 (1)	27 (4)	22 (4)	8 (2)
Decline to answer	4 (< 1)	6 (1)	2 (< 1)	4 (1)	6 (1)	0 (0)
**Educational level, n (%)**						
Less than high school	14 (2)	15 (2)	13 (2)	2 (< 1)	7 (1)	4 (1)
High school	183 (23)	158 (21)	159 (23)	97 (14)	72 (12)	123 (23)
Some college or associate degree	199 (25)	233 (32)	233 (33)	164 (23)	173 (29)	199 (38)
Bachelor’s degree and higher	397 (49)	314 (42)	286 (41)	444 (62)	319 (54)	198 (38)
Other	16 (2)	16 (2)	11 (2)	4 (1)	19 (3)	3 (1)
Decline to answer	3 (< 1)	3 (< 1)	1 (< 1)	1 (< 1)	4 (1)	0 (0)
**Living situation, n (%)**						
Single with child(ren)	56 (7)	46 (6)	70 (10)	94 (13)	101 (17)	134 (25)
Single without child(ren)	177 (22)	237 (32)	215 (31)	-	-	-
Married or partner with child(ren)	354 (44)	244 (33)	215 (31)	610 (86)	468 (79)	383 (73)
Married or partner without child(ren)	195 (24)	182 (25)	179 (25)	-	-	-
Other	26 (3)	25 (3)	22 (3)	7 (1)	21 (4)	9 (2)
Decline to answer	4 (< 1)	5 (1)	2 (< 1)	1 (< 1)	4 (1)	1 (< 1)

Most likely due to the inclusion criteria of having at least one child under the age of 15, most respondents in S2 were aged 30 to 50 years. In the UK and Canada, most respondents were female and had a bachelor’s degree or higher. In the US, most respondents were male and had a bachelor’s degree or higher or some college or an associate degree. The majority in all countries were in full-time employment and were married/living with a partner (Table 3).

### Utilities for people living with haemophilia (S1)

**Fig. 3:**
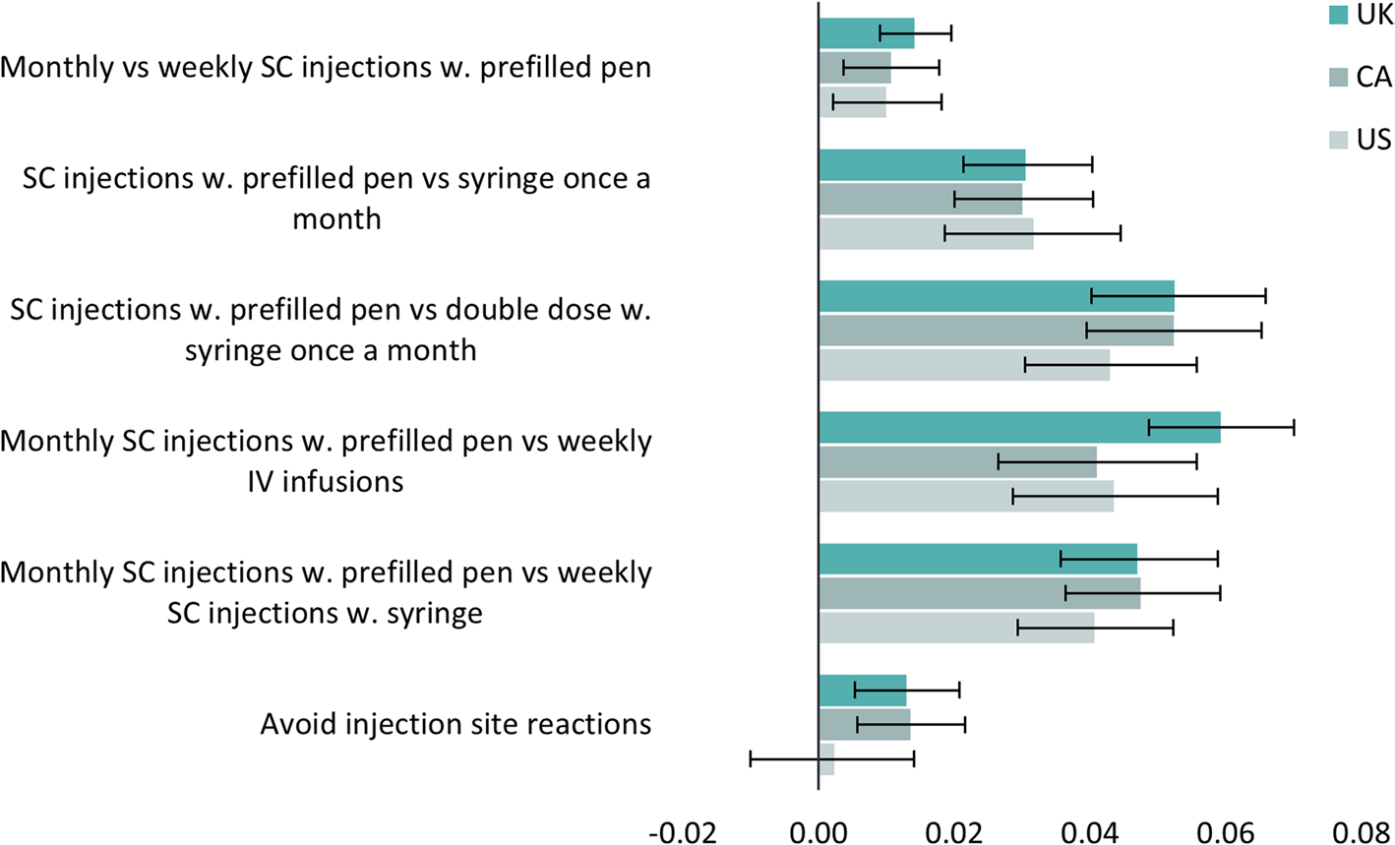
Illustration of utility gains or disutilities associated with different aspects of haemophilia treatment (S1). SC: Subcutaneous, IV: Intravenous

Figure 3 illustrates utility differences associated with the investigated treatment aspects when the general population evaluated health states as if they had haemophilia themselves and were self-administering the treatment.

Among the general population in the UK, Canada and the US, the treatment device was considered the most important aspect. Monthly SC injections with a prefilled pen-device instead of a syringe were associated with a significant yearly utility gain of 0.031 (95% CI: 0.021; 0.040, *p* < 0.001) in the UK, 0.030 (95% CI: 0.020; 0.040, *p* < 0.001) in Canada and 0.032 (95% CI: 0.019; 0.044, *p* < 0.001) in the US. In addition, monthly SC injections with a prefilled pen-device were associated with a significant utility gain compared with monthly double-dose SC injections with a syringe, weekly SC injections with a syringe and IV infusions with a syringe in all three countries.

A lower treatment frequency was also preferred in all countries. Monthly instead of weekly SC injections with a prefilled pen-device were associated with a yearly utility gain of 0.014 (95% CI: 0.009; 0.020, *p* < 0.001) in the UK, 0.011 (95% CI: 0.004; 0.018, *p* = 0.005) in Canada and 0.010 (95% CI: 0.002; 0.018, *p* = 0.011) in the US.

Avoiding injection site reactions was associated with a significant yearly utility gain of 0.013 in both the UK and Canada (95% CI UK: 0.005; 0.021, *p* < 0.001 and 95% CI Canada: 0.006; 0.022, *p* < 0.001). In the US there were no significant results for avoiding injection site reactions.

All results for S1 are presented in Table 4 and Table A5.

**Table 4:** Utility gain/disutility associated with aspects of haemophilia treatment elicited for people living with haemophilia

	**UK**			**Canada**			**US**		
	**N**	**Utility**	**95% CI**	**N**	**Utility**	**95% CI**	**N**	**Utility**	**95% CI**
Monthly vs weekly SC injections w. prefilled pen	269	0.014*	0.009;0.020	261	0.011*	0.004;0.018	229	0.010*	0.002;0.018
SC injections w. prefilled pen vs syringe once a month	234	0.031*	0.021;0.040	239	0.030*	0.020;0.040	201	0.032*	0.019;0.044
SC injections w. prefilled pen vs double dose w. syringe once a month	234	0.052*	0.040;0.066	239	0.052*	0.040;0.065	201	0.043*	0.030;0.056
Monthly SC injections w. prefilled pen vs weekly IV infusions	265	0.059*	0.049;0.070	199	0.041*	0.026;0.056	233	0.043*	0.029;0.059
Monthly SC injections w. prefilled pen vs weekly SC injections w. syringe	234	0.047*	0.036;0.059	239	0.047*	0.036;0.059	201	0.041*	0.029;0.052
Avoid injection site reactions	234	0.013*	0.005;0.021	239	0.013*	0.006;0.022	201	0.002	-0.010;0.014

*SC* Subcutaneous

*IV* Intravenous

^*^P-value < 0.05

### Utilities for caregivers of children with haemophilia (S2)

**Fig. 4:**
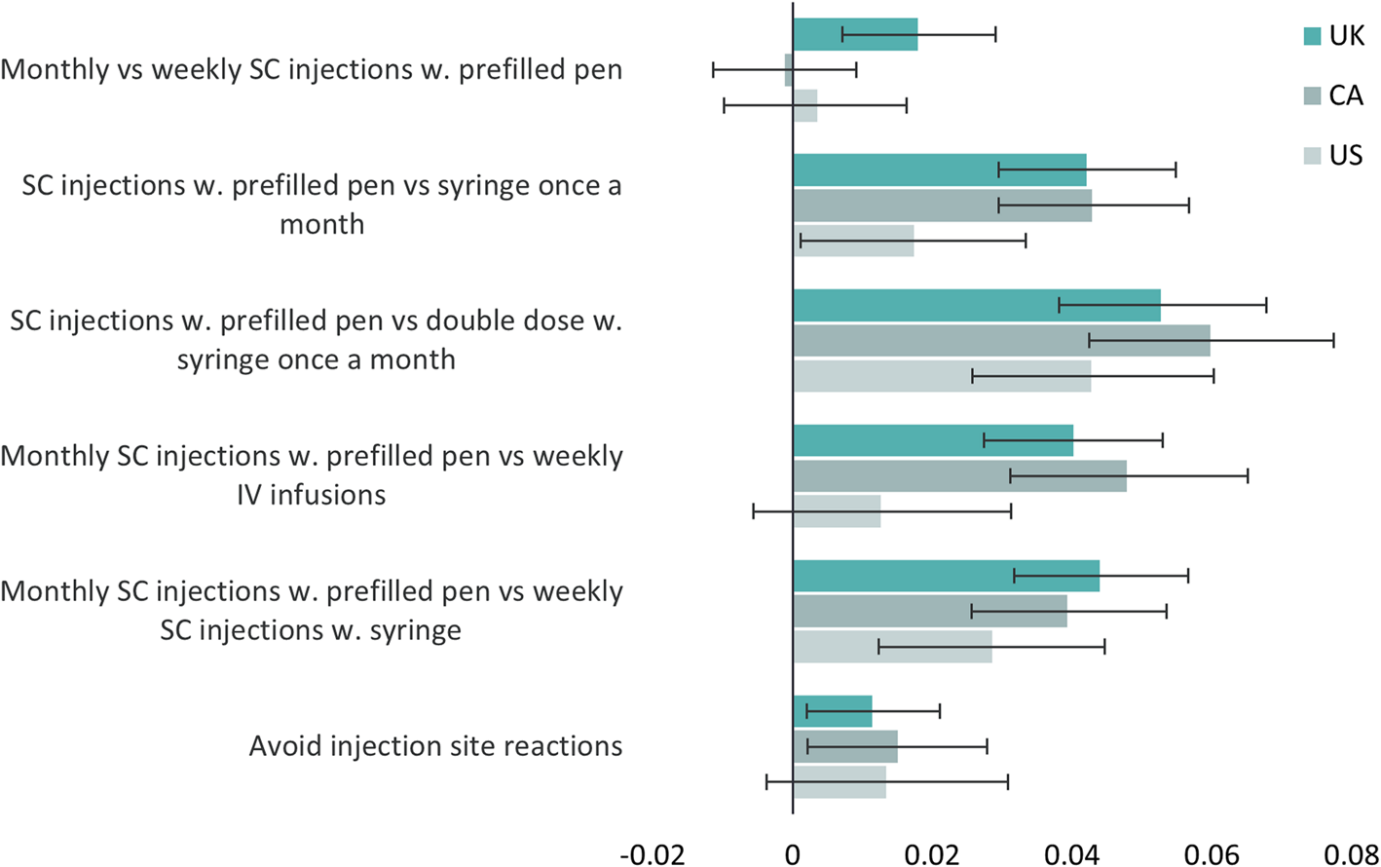
Illustration of utility gains or disutilities associated with different aspects of haemophilia treatment (S2). SC: Subcutaneous, IV: Intravenous

Figure 4 illustrates utility differences associated with the investigated treatment aspects when the general population evaluated health states as if they were treating their child for haemophilia.

In general, the treatment device was the most important aspect in the UK, Canada and the US. Monthly SC injections with a prefilled pen-device instead of a syringe were associated with a significant yearly utility gain of 0.042 (95% CI: 0.030; 0.055, *p* < 0.001) in the UK, 0.043 (95% CI: 0.029; 0.057, *p* < 0.001) in Canada and 0.017 (95% CI: 0.001; 0.033, *p* = 0.037) in the US. Monthly SC injections with a prefilled pen-device were also associated with a significant utility gain when compared with monthly double-dose SC injections with a syringe or weekly SC injections with a syringe in all three countries; however, when compared with weekly IV infusions, they were associated with a significant utility gain only in the UK and Canada.

In S2, avoiding injection site reactions was associated with a significant yearly utility gain of 0.011 (95% CI: 0.002; 0.021, *p* = 0.017) in the UK and 0.015 (95% CI: 0.002; 0.028, *p* = 0.023) in Canada, and lower treatment frequency was associated with a significant yearly utility gain of 0.018 (95% CI: 0.007; 0.029, *p* < 0.001) in the UK only.

All results for S2 are presented in Table 5 and Table A6.

**Table 5:** Utility gain/disutility associated with aspects of haemophilia treatment elicited for caregivers of children with haemophilia

	**UK**			**Canada**			**US**		
	**N**	**Utility**	**95% CI**	**N**	**Utility**	**95% CI**	**N**	**Utility**	**95% CI**
Monthly vs weekly SC injections w. prefilled pen	226	0.018*	0.007; 0.029	201	-0.001	-0.011; 0.009	183	0.004	-0.010; 0.016
SC injections w. prefilled pen vs syringe once a month	222	0.042*	0.030; 0.055	173	0.043*	0.029; 0.057	170	0.017*	0.001; 0.033
SC injections w. prefilled pen vs double dose w. syringe once a month	222	0.053*	0.038; 0.068	173	0.060*	0.042; 0.077	170	0.043*	0.026; 0.060
Monthly SC injections w. prefilled pen vs weekly IV infusions	228	0.040*	0.027; 0.053	188	0.048*	0.031; 0.065	146	0.013	-0.006; 0.031
Monthly SC injections w. prefilled pen vs weekly SC injections w. syringe	222	0.044*	0.032; 0.057	173	0.039*	0.026; 0.054	170	0.028*	0.012; 0.045
Avoid injection site reactions	222	0.011*	0.002; 0.021	173	0.015*	0.002; 0.028	170	0.013	-0.004; 0.031

*SC* Subcutaneous

*IV* Intravenous

^*^P-value < 0.05

## Discussion

Using the TTO method in the UK, Canada and the US, this study estimated utilities associated with haemophilia treatment for people with the disease and potential caregivers. The results emphasise that several treatment aspects have an impact on utilities; however, the level of complexity of the treatment device and the time-use needed for each treatment were associated with the highest utility differences among the three investigated treatment aspects. No major differences between countries were found in the overall results.

In general, treatment with a prefilled pen-device – which is less complex and time-consuming than treatment with a syringe for injections or infusions – was associated with a utility gain for both people with haemophilia and caregivers. This finding aligns with earlier evidence generated using the TTO method. Through interviews with 82 Canadian adults, Johnston et al. (2021) reported that SC prophylactic treatment is associated with higher utilities than those of IV prophylactic treatment [32]. The importance of the treatment device and the time required is further supported by other findings indicating that treatments associated with a high time-use increase the treatment burden [8, 12–16, 33].

Based on the results, less frequent treatments also increase utilities. This finding is in line with the TTO study by Johnston et al. (2021), which reported that an increased treatment frequency results in a decrease in utilities [32]. In addition, a high treatment frequency has been reported as burdensome in a number of other studies, especially when the treatment is administered as an IV infusion, and findings suggest that a reduced infusion frequency is more important than small improvements in efficacy [13–15]. While treatment frequency has an impact on utilities for people living with haemophilia, it seems to have a smaller impact on utilities for caregivers. This is in opposition to previous findings in the literature. Earlier qualitative studies have reported that a reduced treatment frequency can reduce both emotional distress and the practical burden among caregivers [33, 34]. The difference in findings might be explained by the study designs.

The study results further indicate that avoiding injection site reactions in the treatment of haemophilia increases utilities. In this study, only the impact of mild injection site reactions that do not need medical intervention was evaluated (Table 1). Avoiding injection site reactions that have a greater impact on the individual would potentially be associated with even higher utility gains than the ones reported in this study. When compared with findings of a previous migraine study, the utility difference associated with avoiding injection site reactions identified by Matza et al. (2017) is slightly lower [35]. However, it is important to emphasise that caution should be used when drawing comparisons between disease areas. In addition to injection site reactions, results from a recent qualitative study indicate that injection-related pain has an impact on people diagnosed with haemophilia, while other studies have found that a major treatment challenge for caregivers is the amount of pain the procedure causes the child [13, 14, 16, 33]. Considering the fact that pain is already a central factor for people and caregivers living with haemophilia across all severities, treatment options that minimise the amount of injection-related pain are expected to increase utilities for both people living with haemophilia and their caregivers [5, 6, 9].

The results of this TTO study as well as those of previous studies indicate that the current haemophilia treatment is associated with a burden. Therefore, the investigated treatment aspects could be considered in future economic evaluations of haemophilia and haemophilia treatment. The findings emphasise the value of treatment options that are easy to use, minimise the time-use required for administration and reduce the frequency of administration while causing few injection site reactions. Thus, treatment options associated with these features also have the potential to increase HRQoL among both people with haemophilia and caregivers.

Since haemophilia often appears in early childhood, caregivers will be particularly affected when the child is not old enough for self-treatment [1, 28]. The impact of treatment on utilities for caregivers was measured through S2. However, the best method to measure the impact on children diagnosed with haemophilia is unclear, since detailed guidelines on the most appropriate approach in a pediatric population have not yet been developed [23, 36, 37]. Due to the lack of consensus and to maintain consistency in reported measures, the current recommendation is to use the adult general population’s perspective when evaluating children’s HRQoL in economic evaluations [38]. Thus, when discussing the impact of haemophilia treatment on utilities for children, the results from S1 can be considered as a proxy.

The online TTO method applied in this study has both advantages and disadvantages. One disadvantage is that people and caregivers living with haemophilia were not directly involved in the development of the TTO surveys. Instead, surveys were developed based on findings from previous focus group interviews including both people with haemophilia and caregivers as well as insights from medical experts. Thus, all elements included in the surveys were aligned with and validated by experts, which increases the relevance of the study and the validity of the results. Second, the hypothetical nature of the method implies that the TTO surveys were not targeted for people living with haemophilia. Instead, the surveys were targeted for the general population. This approach follows the recommendations of several HTA agencies when eliciting utilities for future health economic evaluations [17–19, 22]. Additionally, the distribution of surveys to the general population made larger sample sizes possible, making the results more robust. To further strengthen the robustness, the most extreme 5% of values were excluded from the analysis. This reduced susceptibility to extreme data points without impacting the findings (Tables A3 and A4). Also, the distribution of the surveys online might have resulted in sample selection bias if the included respondents represented a specific subgroup of the general population. Nevertheless, this risk would also have been present in face-to-face interviews. In addition, the online approach does not provide an opportunity to explain questions further and thereby reduce the potential for misunderstanding. To minimise this limitation, the surveys were developed in collaboration with clinical and economic experts and tested in a pilot study. Additionally, the online approach ensured that the wording remained the same throughout the surveys and minimised the influence of external factors, e.g. interviewer bias. Finally, the online approach reduced social desirability bias through the anonymity of all respondents while making it possible to target specific people and making the final study population as representative as possible.

## Conclusion

This study finds that a number of aspects in haemophilia treatment have an impact on utilities for both people and caregivers living with the disease. Specifically, changes in dosing and administration modalities have a significant impact. In addition, less frequent treatment administrations are expected to further modify this. Thus, these treatment aspects are expected to have an impact on HRQoL among people with haemophilia and potential caregivers.

The results emphasise the potential importance of individualising haemophilia treatment and choosing the most appropriate option for both children and adults living with the disease and their caregivers. The results may inform future health economic analyses of haemophilia and haemophilia treatment as well as future developments in the treatment landscape.

## Supplementary Information

Supplementary Material 1: Appendix. Description of data: Appendix provides additional information about the time trade-off (TTO) method applied in the study, how the treatment aspects were presented for respondents in each survey, and the results of survey 1 (S1) and survey 2 (S2) distributed in the UK, Canada and the US.

## Data Availability

No datasets were generated or analysed during the current study.

## Abbreviations

CI: Confidence interval
HRQoL: Health-related quality of life
HTA: Health technology assessment
IV: Intravenous
SC: Subcutaneous
TTO: Time trade-off
