# Supplementary material for: A time trade-off study in the UK, Canada and the US to estimate utilities associated with the treatment of haemophilia
Source: Health Qual Life Outcomes. 2024 Nov 13;22:97. doi: 10.1186/s12955-024-02311-5 (PMC11558985; doi:10.1186/s12955-024-02311-5)
Supplement: Supplementary file 1 — Supplementary Material 1: Appendix. Description of data: Appendix provides additional information about the time trade-off (TTO) method applied in the study, how the treatment aspects were presented for respondents in each survey, and the results of survey 1 (S1) and survey 2 (S2) distributed in the UK, Canada and the US. [file 12955_2024_2311_MOESM1_ESM.pdf]

Appendix

This appendix provides additional information about the time trade-off (TTO) method applied in the study, how the treatment aspects were presented for respondents in each survey, and the results of survey 1 (S1) and survey 2 (S2) distributed in the UK, Canada and the US.

The time trade-off (TTO) method

Health-related quality of life (HRQoL) associated with specific health states can be evaluated by eliciting utility values. This can be done by using a range of different tools; however, the TTO method is commonly used when measuring the impact of treatment on HRQoL (1).

Using the TTO method, respondents are asked to choose between two health states: 1) an impaired health state for an amount of time, t, or 2) full health but in a shorter amount of life, x. Thus, respondents are asked to "trade" some of their life to live in full health instead of an impaired health state. To estimate utilities associated with the impaired health states, the lifetime for full health, x, is varied until the point of indifference, x*. At this point, the two options are equally attractive to the respondent, and a utility can be calculated as: x*/t. In general, utilities range between 0 (equal to death) and 1 (equal to full health) (2,3).

This study followed a standard bisection methodology with a utility of 0.6 as a starting point to identify the point of indifference. This means that respondents were asked to choose between living x years in the impaired health state and living 0.6 * x years in full health. For a respondent aged about 40 years with an expected remaining lifetime of 40 years, the trade-off would be living in the impaired health state for 40 years and 0 months or living in full health for 24 years and 0 months. Respondents who chose the impaired health state would be asked to trade a smaller number of life years in the next question of the TTO task, while respondents who chose to trade life years to live in full health would be asked to trade a larger number of life years. This would be repeated four to six times to reduce the utility to an interval of 0.025. Since the time horizons included in this study varied for respondents depending on the individual's remaining life expectancy, the maximum number of years possible to trade in the TTO tasks also varied. The minimum amount of life that could be traded was one month. Each respondent's remaining life expectancy was calculated using the most recent lifespan tables from the World Health Organization and the respondent's age and gender (4).

To obtain a better understanding of the respondents' trading behaviour, a follow-up question was presented to the respondents if they were not willing to trade any of their remaining life years or if they were willing to trade the largest possible number of life years. The aim of the follow-up question was to understand the reason for the respondent's choice. Respondents were excluded from the analyses if they reported that ethical or religious beliefs led to their trading behaviour or if they stated that they did not understand the question.

Based on the respondents' trade-offs, an average utility value for each of the nine investigated health states was estimated. Using these utilities, a utility difference between two health states could be calculated and a utility gain or disutility of the three treatment aspects included in this study could be identified. For instance, the only difference between health state 1 and health state 2 in Table 2 is the frequency of administration. Thus, the utility difference between health state 1 and health state 2 can be attributed to the frequency of treatment administration. For this specific example, the utility difference would reflect the impact of a monthly versus a weekly subcutaneous injection using a prefilled pen-device that causes no injection site reactions.

Description of treatment aspects

Some of the description of treatment aspects included in the surveys differed depending on the block, which the respondent was randomised to.

- In block 1, health states 1, 2 and 3 were evaluated.
- In block 2, health states 1, 4, 5, 6 and 7 were evaluated.
- In block 3, health states 1, 5, 8 and 9 were evaluated.

Description of treatment aspects included in survey 1

In the rest of the survey, we ask you to evaluate different treatment options for the bleeding disorder described. For all treatment options, the medicine is taken at your home (or a place of your choice) by yourself. Imagine that before you start the treatment, you will receive thorough instruction and training in how to do it. The treatment itself does not hurt. Imagine that you will need to take the treatment for the rest of your life.

The different treatment options are presented in detail on the next page.

Description included in block 1

The treatment is given with a prefilled, single-use pen device for injection under the skin, which can be stored at room temperature before use. Before the injection, you should wash your hands. The injection process takes approximately 1 minute and consists of the following four steps:

1. Prepare for your injection by cleaning the injection site. Also check that the injection pen is not broken and that the medicine is not expired.
2. Prepare the pen with a new needle and uncap it.
3. Inject the medicine by pressing the pen to your skin and holding the dose button down while you slowly count to 6.
4. After your injection, carefully remove the needle from the pen and throw out the needle and the pen in a sharps disposal container.

The treatment will not cause any injection site reactions after the injection.

The treatment options differ with respect to the frequency of injections you need. The injection can be taken either:

- once every week,
- once every second week, or
- once every month (equal to once every fourth week).

<u>Description included in block 2</u>

The treatment is given as an injection under the skin, but the treatment options differ with respect to the following three elements:

- The administration and storage of the medicine
- The injection site reactions you experience after the treatment
- The frequency of injections

<u>The administration and storage of the medicine</u>

You should always wash your hands before taking the injection. Depending on the treatment option, the administration and storage of the medicine differ. The two available treatment options are:

1. Prefilled, single-use pen device for injection under the skin

   The prefilled, single-use, injection pen can be stored at room temperature. The injection process takes approximately 1 minute and consists of the following four steps:

   a. Prepare for your injection by cleaning the injection site. Also check that the injection pen is not broken and that the medicine is not expired.
   b. Prepare the pen with a new needle and uncap it.
   c. Inject the medicine by pressing the pen to your skin and holding the dose button down while you slowly count to 6.
   d. After your injection, carefully remove the needle from the pen and throw out the needle and the pen in a sharps disposal container.

2. Single-use syringe for injection under the skin

   This option includes a transfer needle, a syringe, and an injection needle. The medicine needs to be stored in the fridge before use. To allow the medicine to reach room temperature, it should be taken out of the refrigerator 15 minutes before the injection.

The injection process takes approximately 5 minutes and consists of the following 16 steps:

a. Remove the cap from the vial with medicine and clean the top of the vial with and alcohol wipe.
b. Attach a transfer needle to the syringe and draw air into the syringe equivalent to your prescribed dose of medicine.
c. Uncap the transfer needle.
d. Inject air into the vial with medicine.
e. Transfer the medicine to the syringe. Fill the syringe with more medicine than your prescribed dose.
f. Remove air bubbles from the medicine by tapping the syringe.
g. Recap the transfer needle.
h. Clean the injection site.
i. Remove the transfer needle from the syringe and throw it out.
j. Attach the injection needle to the syringe.
k. Move the safety shield from the injection needle.
l. Uncap the injection needle.
m. Adjust to your prescribed dose by pushing the plunger.
n. Insert the needle into your skin.
o. Slowly inject the medicine and remove the needle.
p. Cover the needle with the safety shield and throw the needle and the syringe out in a sharps disposal container.

Depending on the specific treatment you need, you may need either one or two injections each time you take this treatment. In case you need two injections, you will need to repeat all of the above steps.

<u>The injection site reactions you experience after the treatment</u>

Depending on the treatment option, the treatment may or may not cause injection site reactions.

- "No injection site reactions" refers to a treatment which never causes any injection site reactions.
- "Injection site reactions" refers to a treatment which always causes injection site reactions within 48 hours after the injection. This includes mild rash, redness of the skin, bruises, itching, or discomfort. No medical intervention such as painkillers or antihistamines will be required to treat the injection site reactions.

<u>The frequency of injections</u>

The injection can be taken with one of the following two frequencies. Either:

- once every week, or
- once every month (equal to once every fourth week).

<u>Description included in block 3</u>

The treatment options differ with respect to the following elements:

- The administration and storage of the medicine

- The frequency of treatment

The administration and storage of the medicine

You should always wash your hands before taking the injection or infusion. Depending on the treatment option, the administration and storage of the medicine differ. The three available treatment options are:

1. Prefilled, single-use pen device for injection under the skin

   The prefilled, single-use, injection pen can be stored at room temperature. The injection process takes approximately 1 minute and consists of the following four steps:

   a. Prepare for your injection by cleaning the injection site. Also check that the injection pen is not broken and that the medicine is not expired.
   b. Prepare the pen with a new needle and uncap it.
   c. Inject the medicine by pressing the pen to your skin and holding the dose button down while you slowly count to 6.
   d. After your injection, carefully remove the needle from the pen and throw out the needle and the pen in a sharps disposal container.

2. Single-use syringe for injection under the skin

   This option includes a transfer needle, a syringe, and an injection needle. The medicine needs to be stored in the fridge before use. To allow the medicine to reach room temperature, it should be taken out of the refrigerator 15 minutes before the injection. The injection process takes approximately 5 minutes and consists of the following 16 steps:

   a. Remove the cap from the vial with medicine and clean the top of the vial with and alcohol wipe.
   b. Attach a transfer needle to the syringe and draw air into the syringe equivalent to your prescribed dose of medicine.
   c. Uncap the transfer needle.
   d. Inject air into the vial with medicine.
   e. Transfer the medicine to the syringe. Fill the syringe with more medicine than your prescribed dose.
   f. Remove air bubbles from the medicine by tapping the syringe.
   g. Recap the transfer needle.
   h. Clean the injection site.
   i. Remove the transfer needle from the syringe and throw it out.
   j. Attach the injection needle to the syringe.
   k. Move the safety shield from the injection needle.
   l. Uncap the injection needle.
   m. Adjust to your prescribed dose by pushing the plunger.
   n. Insert the needle into your skin.
   o. Slowly inject the medicine and remove the needle.
   p. Cover the needle with the safety shield and throw the needle and the syringe out in a sharps disposal container.

3. Single-use syringe for infusion in the vein

   A third option is an IV infusion including a housing containing the medicine, a syringe, and an infusion needle. The medicine can be stored at room temperature before use. The infusion process takes approximately 10 minutes and consists of the following 12 steps:

   a. Prepare for your infusion by checking that the medicine is not expired.
   b. Place the housing on a flat surface with the specified side turning up.
   c. Press down on the housing to mix the medicine.
   d. Swirl gently to dissolve the medicine.
   e. Remove the cap from the housing and connect the syringe. Be careful not to inject air into the medicine.
   f. Turn the housing upside down and draw medicine into the syringe by slowly pulling the plunger back.
   g. Disconnect the syringe from the housing and attach the infusion needle to the syringe.
   h. Remove air bubbles from the medicine by tapping the syringe.
   i. Apply a tourniquet to your arm and clean the injection site.
   j. Insert the needle into the vein and remove the tourniquet. Slowly infuse the medicine for up to five minutes.
   k. Take the needle out of the vein and use sterile gauze to put pressure on the infusion site for several minutes.
   l. Throw the needle and the syringe out in a disposal container.

Irrespective of the treatment option, the treatment will not cause any injection/infusion site reactions after the injection/infusion.

<u>The frequency of treatment</u>

The treatment can be taken with one of the following two frequencies. Either:

- Once every week, or
- once every month (equal to once fourth week).

Description of treatment aspects included in survey 2

In the rest of the survey, we ask you to evaluate different treatment options for the bleeding disorder described. For all treatment options, you give your child the medicine at your home (or a place of your choice). Imagine that before you start the treatment, you will receive thorough instruction and training in how to do it. The treatment itself will not hurt your child.

Imagine that you will need to give your child the treatment for 11 years, from the age of 3 until the age of 14. Beginning at age 14, your child will self-administer the treatment, giving himself or herself a simple injection under the skin every four weeks. This process will take your child a few minutes and causes no injection site reactions. The treatment will keep the disease under control.

The different options for your child’s treatment between the age of 3 until the age of 14 are presented in detail on the following page.

<u>Description of treatment aspects included in block 1</u>

The treatment is given with a prefilled, single-use pen device for injection under the skin, which can be stored at room temperature before use. Before the injection, you should wash your hands. The injection process takes approximately 1 minute and consists of the following four steps:

1. Prepare for your child's injection by cleaning the injection site. Also check that the injection pen is not broken and that the medicine has not expired.
2. Prepare the pen with a new needle and uncap it.
3. Inject the medicine by pressing the pen to your child's skin and holding the dose button down while you slowly count to 6.
4. After the injection, carefully remove the needle from the pen and throw out the needle and the pen in a sharps disposal container.

The treatment will not cause your child any injection site reactions after the injection.

The treatment options differ with respect to the frequency of injections that your child needs. The injection can be given either:

- once every week,
- once every second week, or
- once a month (equal to once every fourth week).

<u>Description of treatment aspects included in block 2</u>

The treatment is given as an injection under the skin, but the treatment options differ with respect to the following three elements:

- Administration and storage of the medicine
- Injection site reactions
- Frequency of injections

<u>Administration and storage of the medicine</u>

You should always wash your hands before giving the injection. Depending on the treatment option, the administration and storage of the medicine differ. The two available treatment options are:

1. Prefilled, single-use pen device for injection under the skin

   The prefilled, single-use injection pen can be stored at room temperature. The injection process takes approximately 1 minute and consists of the following four steps:

   a. Prepare for your child's injection by cleaning the injection site. Also check that the injection pen is not broken and that the medicine has not expired.
   b. Prepare the pen with a new needle and uncap it.
   c. Inject the medicine by pressing the pen to your child's skin and holding the dose button down while you slowly count to 6.
   d. After the injection, carefully remove the needle from the pen and throw out the needle and the pen in a sharps disposal container.

2. Single-use syringe for injection under the skin

   This option includes a transfer needle, a syringe and an injection needle. The medicine needs to be stored in the fridge before use. To allow the medicine to reach room

temperature, it should be taken out of the fridge 15 minutes before the injection. The injection process takes approximately 5 minutes and consists of the following 16 steps:

a. Remove the cap from the vial with medicine and clean the top of the vial with an alcohol wipe.
b. Attach a transfer needle to the syringe and draw air into the syringe equivalent to your child's prescribed dose of medicine.
c. Uncap the transfer needle.
d. Inject air into the vial with medicine.
e. Transfer the medicine to the syringe. Fill the syringe with more medicine than your child's prescribed dose.
f. Remove air bubbles from the medicine by tapping the syringe.
g. Recap the transfer needle.
h. Clean your child's injection site.
i. Remove the transfer needle from the syringe and throw it out.
j. Attach the injection needle to the syringe.
k. Move the safety shield from the injection needle.
l. Uncap the injection needle.
m. Adjust to your child's prescribed dose by pushing the plunger.
n. Insert the needle into your child's skin.
o. Slowly inject the medicine and then remove the needle.
p. Cover the needle with the safety shield and throw out the needle and the syringe out in a sharps disposal container.

Depending on the specific treatment your child needs, you may have to give either one or two injections each time you give your child this treatment. In case your child needs two injections, you will need to repeat all the above steps.

<u>Injection site reactions</u>

Depending on the treatment option, your child may or may not experience injection site reactions.

- "No injection site reactions" refers to a treatment which never causes any injection site reactions.
- "Injection site reactions" refers to a treatment which always causes injection site reactions within 48 hours after the injection. Reactions include mild rash, redness of the skin, bruises, itching, or discomfort. No medical intervention such as painkillers or antihistamines will be required to treat the injection site reactions.

<u>Frequency of injections</u>

The injection can be given with one of the following two frequencies. Either:

- once every week, or
- once a month (equal to once every fourth week).

<u>Description of treatment aspects included in block 3</u>

The treatment options differ with respect to the following elements:

- Administration and storage of the medicine

- Frequency of treatment

Administration and storage of the medicine

You should always wash your hands before giving the injection or infusion. Depending on the treatment option, the administration and storage of the medicine differ. The three available treatment options are:

1. Prefilled, single-use pen device for injection under the skin

   The prefilled, single-use injection pen can be stored at room temperature. The injection process takes approximately 1 minute and consists of the following four steps:

   a. Prepare for your child's injection by cleaning the injection site. Also check that the injection pen is not broken and that the medicine has not expired.
   b. Prepare the pen with a new needle and uncap it.
   c. Inject the medicine by pressing the pen to your child's skin and holding the dose button down while you slowly count to 6.
   d. After the injection, carefully remove the needle from the pen and throw out the needle and the pen in a sharps disposal container.

2. Single-use syringe for injection under the skin

   This option includes a transfer needle, a syringe and an injection needle. The medicine needs to be stored in the fridge before use. To allow the medicine to reach room temperature, it should be taken out of the fridge 15 minutes before the injection. The injection process takes approximately 5 minutes and consists of the following 16 steps:

   a. Remove the cap from the vial with medicine and clean the top of the vial with an alcohol wipe.
   b. Attach a transfer needle to the syringe and draw air into the syringe equivalent to your child's prescribed dose of medicine.
   c. Uncap the transfer needle.
   d. Inject air into the vial with medicine.
   e. Transfer the medicine to the syringe. Fill the syringe with more medicine than your child's prescribed dose.
   f. Remove air bubbles from the medicine by tapping the syringe.
   g. Recap the transfer needle.
   h. Clean your child's injection site.
   i. Remove the transfer needle from the syringe and throw it out.
   j. Attach the injection needle to the syringe.
   k. Move the safety shield from the injection needle.
   l. Uncap the injection needle.
   m. Adjust to your child's prescribed dose by pushing the plunger.
   n. Insert the needle into your child's skin.
   o. Slowly inject the medicine and then remove the needle.
   p. Cover the needle with the safety shield and throw out the needle and the syringe out in a sharps disposal container.

3. Single-use syringe for infusion in the vein

A third option is an IV infusion which involves using a housing containing the medicine, a syringe, and an infusion needle. The medicine can be stored at room temperature before use. The infusion process takes approximately 10 minutes and consists of the following 12 steps:

a. Prepare for your child's infusion by checking that the medicine has not expired.
b. Place the housing on a flat surface with the specified side turned up.
c. Press down on the housing to mix the medicine.
d. Swirl gently to dissolve the medicine.
e. Remove the cap from the housing and connect the syringe. Be careful not to inject air into the medicine.
f. Turn the housing upside down and draw medicine into the syringe by slowly pulling back the plunger.
g. Disconnect the syringe from the housing and attach the infusion needle to the syringe.
h. Remove air bubbles from the medicine by tapping the syringe.
i. Apply a tourniquet to your child's arm and clean the injection site.
j. Insert the needle into your child's vein and remove the tourniquet. Slowly infuse the medicine for up to 5 minutes.
k. Take the needle out of the vein and use sterile gauze to put pressure on the infusion site for several minutes.
l. Throw out the needle and the syringe out in a sharps disposal container.

Irrespective of the treatment option, the treatment will not cause your child any injection or infusion site reactions after the injection or infusion.

<u>Frequency of treatment</u>

The treatment can be given with one of the following two frequencies. Either:

- once every week, or
- once every month (equal to once every fourth week).

Example of health state description in long and short forms

When presented with a new health state, respondents were first presented with a long description of the health state. Subsequently, when asked to choose between the impaired health state and full health, respondents were presented with a shorter description of the health state. Table A1 and Table A2 present examples of how health states were presented in a long and short forms.

Table A1: Long and short descriptions of health state 1 in survey 1

Long description	Short description
We would now like you to imagine that you have the bleeding disorder as described.<br>The disease requires that you give yourself an injection under the skin once every fourth week.<br>The injection is given with a prefilled injection pen device, which can be stored at room temperature.<br>The injection process takes around one minute and requires four steps.<br>The treatment causes no injection site reactions.<br>The treatment keeps the disease under control.<br>Imagine what it would be like for you to live with this disease every day for the rest of your life.	You give yourself an injection in the skin once every fourth week, and you use a prefilled pen device.<br>The injection takes around one minute and requires four steps.<br>The treatment causes no injection site reactions.

Table A2: Long and short descriptions of health state 1 in survey 2

Long description	Short description
We would now like you to imagine that you have a 3-year-old child with the bleeding disorder described.<br>The disease requires that you give your child an injection under the skin once every fourth week.<br>You give the injection with a prefilled injection pen device, which can be stored at room temperature.<br>The injection process takes around one minute and requires four steps.<br>The treatment causes no injection site reactions for your child.<br>The treatment keeps your child's disease under control.<br>Imagine what it would be like for you to give your child the treatment for 11 years.	You give your 3-year-old child an injection in the skin once every fourth week for 11 years, using a prefilled injection pen device.<br>The injection takes around one minute and requires four steps.<br>The treatment causes no injection site reactions for your child.

Results of sensitivity analysis

To investigate the impact of excluding the most extreme 5% of values from the data analysis, a sensitivity analysis, in which all values were included, was conducted. The results are presented in Table A3 and Table A4.

Table A3: Sensitivity analysis of S1 (people living with haemophilia)

	UK			Canada			US		
	**N**	**Utility**	**95% CI**	**N**	**Utility**	**95% CI**	**N**	**Utility**	**95% CI**
Monthly vs weekly SC injections w. prefilled pen	285	0.020*	0.010;0.032	275	0.011*	0.001;0.021	243	0.016*	0.000;0.033
SC injections w. prefilled pen vs syringe once a month	248	0.033*	0.019;0.047	253	0.029*	0.013;0.045	213	0.039*	0.021;0.059
SC injections w. prefilled pen vs double dose w. syringe once a month	248	0.060*	0.042;0.080	253	0.056*	0.039;0.075	213	0.045*	0.028;0.061
Monthly SC injections w. prefilled pen vs weekly IV infusions	279	0.066*	0.052;0.081	211	0.043*	0.023;0.064	247	0.046*	0.026;0.068
Monthly SC injections w. prefilled pen vs weekly SC injections w. syringe	248	0.049*	0.032;0.066	253	0.051*	0.034;0.068	213	0.042*	0.026;0.058
Avoid injection site reactions	248	0.014*	0.002;0.027	253	0.019*	0.005;0.035	213	-0.005	-0.027;0.014

*Statistically significant result.

Table A4: Sensitivity analysis of S2 (caregivers of children with haemophilia)

	UK			Canada			US		
	**N**	**Utility**	**95% CI**	**N**	**Utility**	**95% CI**	**N**	**Utility**	**95% CI**
Monthly vs weekly SC injections w. prefilled pen	238	0.024*	0.008;0.040	213	0.003	-0.013;0.020	193	0.008	-0.010;0.027
SC injections w. prefilled pen vs syringe once a month	234	0.045*	0.028;0.063	183	0.050*	0.031;0.071	180	0.016	-0.008;0.040
SC injections w. prefilled pen vs double dose w. syringe once a month	234	0.059*	0.039;0.080	183	0.070*	0.047;0.095	180	0.046*	0.022;0.072
Monthly SC injections w. prefilled pen vs weekly IV infusions	240	0.042*	0.024;0.060	198	0.053*	0.032;0.076	154	0.017	-0.006;0.042
Monthly SC injections w. prefilled pen vs weekly SC injections w. syringe	234	0.047*	0.030;0.065	183	0.048*	0.028;0.069	180	0.023*	0.000;0.044
Avoid injection site reactions	234	0.014	-0.002;0.029	183	0.016	-0.001;0.034	180	0.014	-0.010;0.038

*Statistically significant result.

Additional results of the TTO surveys

Utility differences presented in Table 4 and Table 5 were assessed to be the most relevant results for future health economic evaluations of haemophilia treatment. However, based on the nine health states investigated in the surveys, additional utility differences were elicited. These results provide further details on the impact of haemophilia treatment on utilities for people with the disease and potential caregivers. Additionally, the results can be included in future health economic evaluations if relevant.

Table A5 presents additional utility gains/disutilities measured using S1, and Table A6 presents additional utility gains/disutilities measured using S2.

Table A5: Additional results elicited for people living with haemophilia using S1

	UK			Canada			US		
	**N**	**Utility**	**95% CI**	**N**	**Utility**	**95% CI**	**N**	**Utility**	**95% CI**
Monthly vs biweekly SC injections w. prefilled pen	269	0.006*	0.002; 0.010	261	0.005	-0.001; 0.011	229	0.003	-0.005; 0.011
Biweekly vs weekly SC injections w. prefilled pen	269	0.010*	0.004; 0.015	261	0.005	-0.002; 0.012	229	0.009	-0.0003; 0.017
SC injections w. syringe vs double dose with syringe once a month	234	0.023*	0.015; 0.033	239	0.022*	0.014; 0.031	201	0.015*	0.002; 0.028
Monthly vs weekly SC injections w. syringe	234	0.012*	0.004; 0.020	239	0.017*	0.011; 0.024	201	0.009	-0.002; 0.020
SC injections w. prefilled pen vs IV infusions once a month	265	0.052*	0.042; 0.063	199	0.021*	0.010; 0.033	233	0.030*	0.017; 0.042
SC injections w. syringe vs IV infusions once a month	265	0.007	-0.0004; 0.015	199	-0.004	-0.013; 0.005	233	0.002	-0.009; 0.014

*P-value <0.05
SC: Subcutaneous
IV: Intravenous

Table A6: Additional results elicited for caregivers of children with haemophilia using S2

	UK			Canada			US		
	**N**	**Utility**	**95% CI**	**N**	**Utility**	**95% CI**	**N**	**Utility**	**95% CI**
Monthly vs biweekly SC injections w. prefilled pen	226	0.021*	0.010; 0.033	201	0.001	-0.010; 0.012	183	-0.009	-0.021; 0.004
Biweekly vs weekly SC injections w. prefilled pen	226	0.0001	-0.011; 0.011	201	-0.002	-0.012; 0.009	183	0.016*	0.003; 0.028
SC injections w. syringe vs double dose with syringe once a month	222	0.011*	0.002; 0.020	173	0.018*	0.005; 0.031	170	0.025*	0.008; 0.042
Monthly vs weekly SC injections w. syringe	222	0.001	-0.008; 0.009	173	-0.004	-0.014; 0.007	170	0.008	-0.005; 0.023
SC injections w. prefilled pen vs IV infusions once a month	228	0.024*	0.013; 0.035	188	0.014*	0.002; 0.027	146	0.009	-0.006; 0.025
SC injections w. syringe vs IV infusions once a month	228	0.006	-0.004; 0.015	188	-0.006	-0.017; 0.005	146	-0.013	-0.030; 0.004

*P-value <0.05
SC: Subcutaneous
IV: Intravenous

References

1. Matza LS, Stewart KD, Lloyd AJ, Rowen D, Brazier JE. Vignette-Based Utilities: Usefulness, Limitations, and Methodological Recommendations. Value in Health. 2021 Jun;24(6):812–21.

2. Whitehead SJ, Ali S. Health outcomes in economic evaluation: the QALY and utilities. British Medical Bulletin. 2010 Dec 1;96(1):5–21.

3. Torrance GW, Feeny D. Utilities and Quality-Adjusted Life Years. International Journal of Technology Assessment in Health Care. 1989;5(04):559–75.

4. Life tables for WHO member states [Internet]. Available from: https://apps.who.int/gho/data/node.main.687?lang=en
